# Quality indicators for blood culture: 1 year of monitoring with BacT/Alert Virtuo at a French hospital

**DOI:** 10.1099/jmm.0.001300

**Published:** 2020-12-17

**Authors:** Cécile Emeraud, Seher Yilmaz, Nicolas Fortineau, Gaëlle Cuzon, Laurent Dortet

**Affiliations:** ^1^​ Department of Bacteriology-Hygiene, Bicêtre Hospital, Assistance Publique - Hôpitaux de Paris, Le Kremlin-Bicêtre, France; ^2^​ UMR-S 1184, Paris-Saclay University, Le Kremlin-Bicêtre, France; ^3^​ French National Reference Center for Antibiotic Resistance, France; ^4^​ Paris-Saclay University, Faculty of Medicine, Le Kremlin-Bicêtre, France

**Keywords:** accreditation, BacT/AlertVirtuo, blood culture, quality indicators

## Abstract

**Introduction:**

Blood culture (BC) remains the gold standard for the diagnosis of bloodstream infection. Clinical microbiology laboratories must ensure the quality of their BC process from receipt to definitive results.

**Aim:**

In this study, we followed the evolution of different quality indicators for BCs over the first year of implementation of the BacT/Alert Virtuo system in a French hospital.

**Methodology:**

In our laboratory, we instituted regular monitoring of several quality indicators to track (i) delays in sample registration, (ii) delays in loading BC bottles in our incubating system (BacT/Alert Virtuo) after registration, (iii) the volume of blood in bottles and (iv) the contamination rates.

**Results:**

For 53 892 BC bottles loaded in the BacT/Alert Virtuo from 23 January to 31 December 2019, the delays in sample registration, loading and unloading were respectively 3.5 h±0.016, 44 min±0.209 and 5.8 h±0.0727. Intriguingly, the automated process performed by the BacT/Alert Virtuo system to check the blood volume in bottles was only performed for 60 % of the loaded bottles. Among these, 30 % contained the recommended volume of blood (between 7 and 13 ml). Finally, the contamination rate was found to be 27.2 % for samples at our institution.

**Conclusions:**

The delays in sample registration, loading and unloading were found to be acceptable, even though they could be improved by ensuring a continuous service during the night duty period. Furthermore, the percentage of volumes measured is insufficient and must be improved and the majority of bottles do not contain the recommended blood volume.

## Introduction

Bloodstream infections are associated with high mortality and morbidity worldwide [1, 2]. Blood culture (BC) is essential for the diagnosis of sepsis. It remains the gold standard for the detection and culture of the micro-organisms involved in the bacteraemia before antimicrobial susceptibility testing. The delay between BC sampling and definitive results should be as short as possible to implement the most appropriate antimicrobial therapy [3]. BacT/Alert Virtuo (bioMérieux, Durham, NC, USA) is the latest version of the automated bioMérieux BC system that facilitates rapid detection of positive BCs [4, 5]. On top of its automated loading and unloading process for the BC bottles, this system is able to perform measurement of blood volume by scanning the sample level of each bottle with a camera. This last technology is crucial, since blood volume has been demonstrated to be one of the most critical factors for successful results [6]. Accordingly, the optimal volume of blood per bottle must be between 7 and 13 ml. If this volume is insufficient, there is a statistical increase in the risk of a false-negative result [7].

Within the framework of BC accreditation described by the international standard ISO 15189, quality indicators are fundamental tools for accuracy and patient safety [8, 9]. These indicators facilitate the monitoring and evaluation of laboratory performance across critical points in pre-examination, examination and post-examination processes. In this study, we followed the evolution of different quality indicators for BCs over the first year of implementation of the BacT/Alert Virtuo system in a French hospital.

## Methods

### Hospitals

Bicêtre Hospital (BCT) belongs to the Paris-Sud University Hospitals group, which is one of the 12 hospital groups of the Assistance Publique – Hôpitaux de Paris (AP-PH). It is a 1000-bed tertiary teaching hospital with a large number of medical and surgical specialties. The clinical laboratory of bacteriology at Bicêtre also receives samples from Paul Brousse Hospital (PBR), an 800-bed tertiary teaching university hospital. Samples from BCT are received by the laboratory throughout the day and night, and those from PBR are delivered several times per day by couriers between 7 a.m. and 7 p.m. During the night duty period (Monday–Saturday every day lasts from 7 p.m. to 7 a.m., but on Sunday this is reduced to the afternoon), with a reduced technical staff, positive BC bottles are not unloaded from the automated system.

### BC system

Since January 2019, the laboratory has been equipped with a BacT/Alert Virtuo (bioMérieux, Durham, NC, USA) for blood infection detection. Three types of BC bottles are used: (i) aerobic bottles (BacT/Alert FA plus), (ii) anaerobic bottles (BacT/Alert FN plus) and (iii) paediatric bottles (BacT/Alert PF plus).

### Followed indicators

Within the framework of BC accreditation, the following quality indicators were chosen for monitoring: (i) number of BC bottles received per month, (ii) delay from sampling to registration (=routing delay), (iii) delay from registration to loading of the bottles in the incubation system (=loading delay), (iv) positive bottle unloading delay (=unloading delay), (v) positive bottle rate, (vi) percentage of bottles for which the volumes are automatically measured, (vii) percentage of bottles with conforming volume (7 to 13 ml for FA and FN plus bottles), (viii) BacT/Alert Virtuo false-positivity rate and (ix) rate of BC sample contamination.

All indicators, except the rate of contamination and false positives per BC bottle, were extracted each month from the middleware MYLA (bioMérieux, Durham, NC, USA) and entered into an Excel workbook, allowing automatic calculation of all indicators. The rate of false-positive growth signals that corresponds to the percentage of bottles with a positive signal and a negative culture was extracted every 15 days from the BacT/Alert Virtuo.

Volumes were considered to be non-conforming if they measured less than 7 ml or more than 13 ml. The collection of these data began on 23 January 2019, corresponding to the date of the implementation of the BacT/Alert Virtuo system in our laboratory.

The BC contamination rate was evaluated per sample (aerobic and anaerobic bottles correspond to one sample for adults and the paediatric aerobic bottle corresponds to one sample for children). Data were extracted from the laboratory information management system (GLIMS edited by MIPS, Vincennes, France). A unique positive BC sample with recognized contaminants (coagulase-negative staphylococci, *Micrococcus* spp., *Bacillus* spp. and *viridans* group streptococci) was considered as probable contamination [10].

## Results

A total of 53 892 BC bottles were introduced into the BacT/Alert Virtuo from 23 January to 31 December 2019. Of these, 95 % were aerobic or anaerobic bottles and 5 % were paediatric bottles (Table 1). The monthly number of bottles received was stable at 4769±118 (average±sem), except for a drop in activity in August (*n*=3881 bottles), corresponding to the summer school holidays (Table 1).

**Table 1.** Monitoring of quality indicators for blood cultures for 2019

		No. of bottles				Delay (mean)			No. of positives	Virtuo false-positivity rate									
		Total	FA	FN	PF	Reception	Loading in Virtuo	Unloading in Virtuo		No. of false positives*	No. of false positives*/ total (%)	No. of false positives*/ total positives (%)	Measured bottles (FN, FA)	Low volumes <7 ml	High volumes >13 ml	Conforming volumes† (among measured)	Non-conforming volumes† (among measured)	Conforming volumes † (total)	Non-conforming volumes † (total)
**January**	*n*	1436	688	686	62	4.3 h	40 min	7.35 h	148	0			625	419	35	171	454	171	454
	%		**48**	**48**	**4**				**10**		**0**	**0**	**44**	**29**	**2**	**27**	**73**	**12**	**32**
**February**	*n*	5159	2485	2475	199	3.7 h	43 min	5.5 h	524	2			3277	2117	280	880	2397	880	2397
	**%**		**48**	**48**	**4**				10		**0.04**	**0.38**	**64**	**41**	**5**	**27**	**73**	**17**	**46**
**March**	*n*	5300	2528	2529	243	3.3 h	45 min	5.8 h	576	3			3367	2212	246	909	2458	909	2458
	%		**47.5**	**47.5**	**5**				11		**0.06**	**0.52**	**64**	**42**	**5**	**27**	**73**	**17**	**46**
**April**	*n*	5009	2390	2388	231	3.6 h	49 min	5.8 h	538	4			3006	2007	244	755	2251	755	2251
	%		**47.5**	**47.5**	**5**				11		**0.08**	**0.74**	**60**	**40**	**5**	**25**	**75**	**15**	**45**
**May**	*n*	4827	2310	2307	210	3.9 h	47 min	6.0 h	432	3			2827	1759	284	784	2043	784	2043
	%		**48**	**48**	**4**				9		**0.06**	**0.69**	**59**	**36**	**6**	**28**	**72**	**16**	**42**
**June**	*n*	4843	2304	2299	240	3.9 h	47 min	5.3 h	542	2			2891	1631	317	943	1948	943	1948
	%		**47.5**	**47.5**	**5**				11		**0.04**	**0.37**	**60**	**34**	**7**	**33**	**67**	**19**	**40**
**July**	*n*	4739	2270	2270	199	3.1 h	39 min	5.8 h	556	0			2426	1494	210	722	1704	722	1704
	%		**48**	**48**	**4**				12		**0.00**	**0.00**	**51**	**32**	**4**	**30**	**70**	**15**	**36**
**August**	*n*	3881	1827	1820	234	2.7 h	37 min	5.8 h	474	0			2223	1351	190	682	1541	682	1541
	%		**47**	**47**	**6**				12		**0.00**	**0.00**	**57**	**35**	**5**	**31**	**69**	**18**	**40**
**September**	*n*	4367	2067	2071	229	3.1 h	44 min	4.2 h	457	5			**2727**	**1669**	**270**	**788**	**1939**	**788**	**1939**
	%		**47.5**	**47.5**	**5**				10		**0.11**	**1.09**	**62**	**38**	**6**	**29**	**71**	**18**	**44**
**October**	*n*	4926	2327	2326	273	3.6 h	43 min	6.0 h	528	9			3416	2096	354	966	2450	966	2450
	%		**47**	**47**	**6**				11		**0.18**	**1.70**	**69**	**43**	**7**	**28**	**72**	**20**	**50**
**November**	*n*	4837	2288	2289	260	3.8 h	48 min	6.3 h	493	2			3321	1991	354	976	2345	976	2345
	%		**47.5**	**47.5**	**5**				10		**0.04**	**0.41**	**69**	**41**	**7**	**29**	**71**	**20**	**48**
**December**	*n*	4568	2144	2144	280	3.6 h	44 min	6.4 h	504	5			2954	1760	337	857	2097	857	2097
	%		**47**	**47**	**6**				11		**0.11**	**0.99**	**65**	**39**	**7**	**29**	**71**	**19**	**46**
**Total**	*n*	53 892	25 628	25 604	**2660**	3.5 h	44 min	5.8 h	5772	35			33 060	20 506	3121	9433	23 627	9433	23 627
	%		**47.5**	**47.5**	**5**				11		**0.07**	**0.61**	**60**	**37**	**6**	**30**	**70**	**17**	**43**

∗ False positive, bottles with positive signal with BacT/Alert Virtuo and negative culture.

†Conforming volumes, volumes between 7 and 13 ml.

nd:,not determined; FA, aerobic bottles; FN, anaerobic bottles; PF, paediatric bottles.

**Fig. 1.**
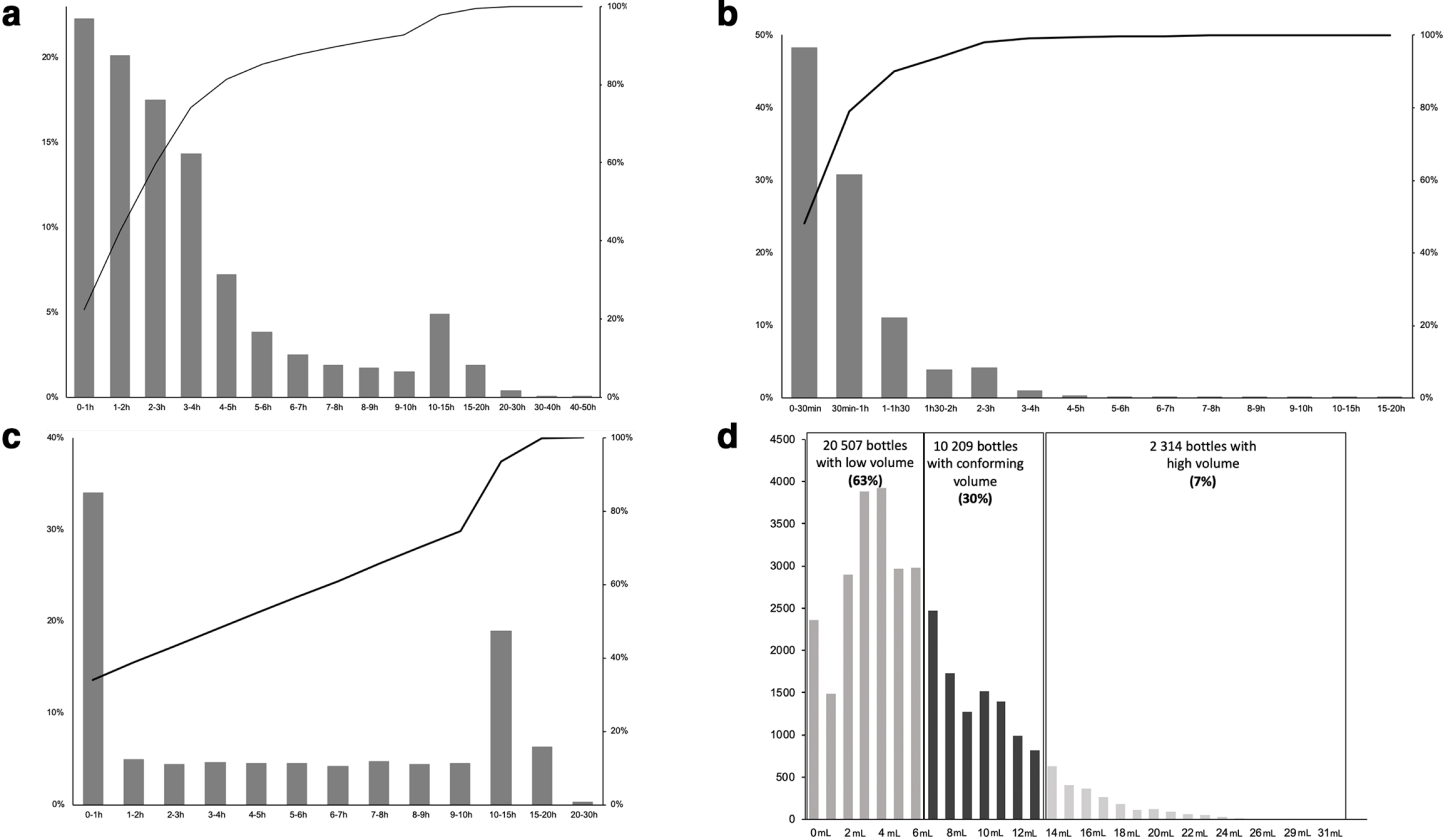
Quality indicators for blood culture bottles using the BacT/Alert Virtuo over 1 year: (**a**) delay from sampling to registration (=routing delay), (**b**) delay from sample registration to loading (=loading delay), (**c**) delay to unloading positive bottle (=unloading delay) and (**d**) percentage and distribution of measured blood volumes in bottles.

For all BCs, the average delay between sampling in the clinical ward and sample registration in the laboratory (=routing delay) was 3.5 h±0.016 (Table 1). The median routing delay was 2.5 h, and 5 % of samples had a routing delay longer than 12 h (Fig. 1a). During the day (7 a.m. to 7 p.m.), the average routing delay was 3.1 h±0.021. This delay significantly (*P* <0.001, Student’s *t*-test) increased to 4.2 h±0.026 during night duty periods. Since the bacteriology laboratory is located in BCT, this routing delay is significantly quicker for samples from BCT than for those from PBR located at 2 km (2.6 h±0.0206 vs 6.5 h±0.0686, *P* <0.001).

Regarding the delay between loading bottles into the BacT/Alert Virtuo and registration in the laboratory information management system (=loading delay), the average time was 44 min ±0.209 and the median was 30 min (Table 1). A difference could be observed between day and night duty periods (44 min±0.0032 vs 46 min±0.0090 average delay, *P* <0.001). The majority of the bottles (94.1 %) were loaded into the automated system in less than 2 h (Fig. 1b).

Regarding the delay in unloading positive bottles (=unloading delay), the average time was 5.8 h±0.0727 and the median was 4.5 h (Table 1). Interestingly, the distribution of unloading delays identified two peaks (Fig. 1c) at less that 1 h (33.9 % of positive BCs) and between 10 and 15 h (19.0 % of positive BCs). As expected, the positive BC bottles unloaded in less than 1 h likely correspond to those that were positive during the day duty period, whereas positive BC bottles unloaded between 10h to 15h correspond to those that were positive during the night duty period.

Of note, the blood volume was only measured automatically by the BacT/Alert Virtuo for 60 % of the loaded bottles (FA and FN plus bottles) (Table 1). During the first month’s use of the BacT/Alert Virtuo the volumes were only measured for 44 % of the loaded bottles, whereas this rate reached almost 70 % by the end of the year (Table 1). Among the bottles for which the volume was measured, only 30 % had a conforming volume (7 to 13 ml). These volumes were less than 7 ml or more than 13 ml in 63 and 7 % of the cases, respectively (Fig. 1d).

**Fig. 2.**
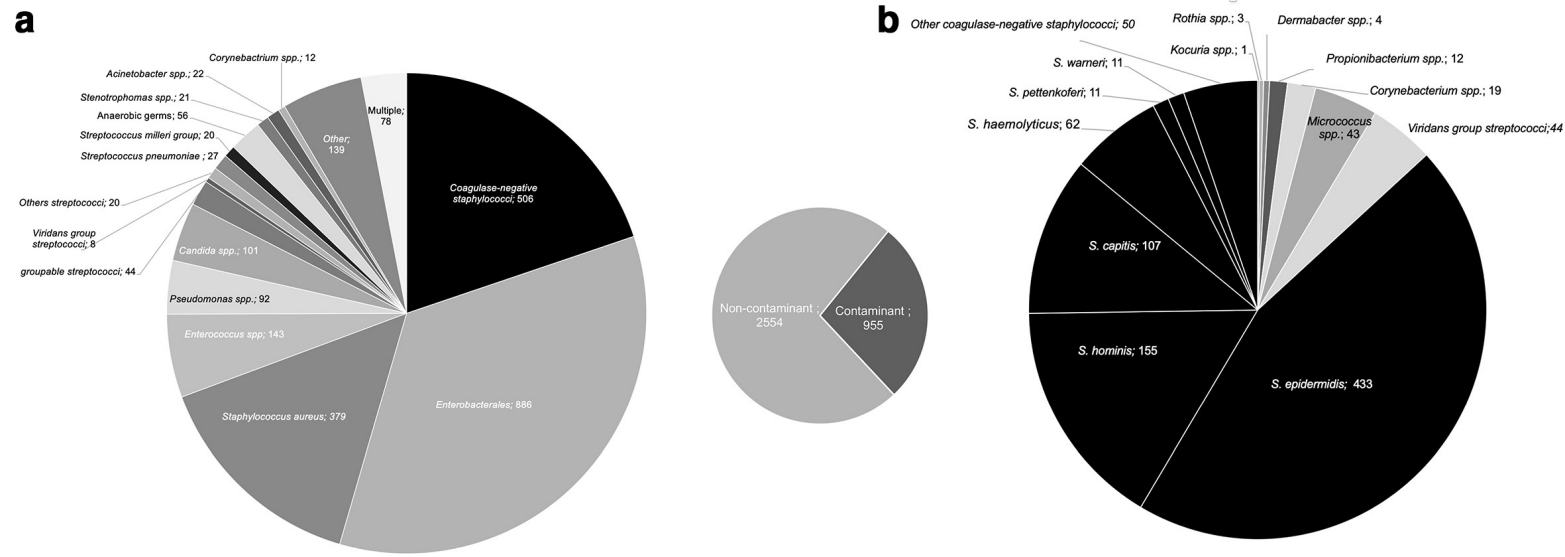
Blood culture contamination over 1 year: (**a**) identification of germs grown in positive blood culture samples and (**b**) rate and identification of contaminants in positive blood cultures samples.

Finally, 11 % (5772/53 892) of the loaded BC bottles turned to positive (Table 1), with this number corresponding to 3509 BC samples. Among these positive BC bottles, 35 (0.67 %) were actually negative in culture (Table 1) and thus considered to be ‘false positives’. Among the 3509 BC samples with positive bottles, 72.8 % (2554/3509) were positive with germs that were considered to be responsible for bacteraemia (Fig. 2). The most prevalent organisms responsible for ‘true’ bacteraemia are Enterobacterales (34.7 %, *n*=886), coagulase-negative *Staphylococcus* (19.1 %, *n*=506), *Staphylococcus aureus* (14.8 %, *n*=379) and *Enterococcus* spp. (5.6 %, *n*=143) (Fig. 2, left panel). Among these BCs, 3.1 % (*n*=78) were positive with at least two different germs. The last 955 (272 %) positive BC samples led to the identification of contaminants (Fig. 2). Contaminated cultures mostly correspond to the growth of coagulase-negative staphylococci (86.8 %, *n*=829), including a majority of *Staphylococcus epidermidis* (*n*=433), *Staphylococcus hominis* (*n*=155) *S. capitis* (*n*=107) and *Staphylococcus haemolyticus* (*n*=62). The other contaminants corresponded to *viridans* group streptococci (*n*=44), *Micrococcus* spp. (*n*=43), *Corynebacterium* spp. (*n*=19), *Propionibacterium* spp. (*n*=12), *Dermabacter* spp. (*n*=4), *Rothia* spp. (*n*=3) and *Kocuria* spp. (*n*=1) (Fig. 2, right panel).

## Discussion

Monthly monitoring of quality indicators facilitates the implementation of improvements and rapidly identifies variations that might have a clinical or a logistical impact. First, we observed that a large proportion (5.1 %) of bottles took longer than 12 h to be transported from the clinical ward to the laboratory. These were primarily bottles coming from PBR. Indeed, samples from PBR are delivered several times a day by couriers, but they are not delivered during the night duty period. Accordingly, it might be helpful to increase the rotation in order to limit this delay. Regarding the delay between registration in the laboratory management system and the loading of the BC bottles into the BacT/Alert Virtuo (=loading delay), the average time is very satisfactory (44 min±0.209) independently of the duty period. The large majority (94.0 %) of the bottles were loaded in less than 2 h. In addition, the loading delay (44 min±0.209) seems to be negligible compared to the routing delay (3.5 h±0.016), limiting any possible improvement process. Finally, the mean delay in unloading positive bottles is 5.8 h±0.0727. This relatively long delay is explained by the fact that the positive bottles are not unloaded during the night duty period. Accordingly, BC that turn positive at the end of the day will not be analysed before the morning, resulting in an increase in the average and median unloading delay. Of note, a peak of BCs unloaded in less than 1 h (33.9 %) can be observed. This corresponds to BCs that turn positive during day duty. Consequently, the unloading delay could be improved if the management of positive BCs were the same during night and day duty period. However, this implies huge organizational changes, which must be taken in account.

Since the clinical impact of adequate volume of blood inside bottles is well known, use of a system that is able to measure the collected blood volumes seems to be a real advantage. Indeed, it has been shown that the higher the volume of BCs, the higher the rate of detection of bloodstream infections [7]. According to guidelines, the targeted blood volume should be between 8 and 10 ml per bottle. However, BacT/Alert Virtuo system is not accurate to within 2 ml, explaining the range set up (7–13 ml). However, our study highlights two major issues. First, the blood volume is only measured for 60 % of the loaded bottles. Thus, we cannot assess whether the blood volume is adequate in bottles that are not measured. Second, only 30 % of the measured volumes were conforming (between 7 and 13 ml), and a large majority of the bottles (63 %) contained less than 7 ml of blood. The improvement in reading between January and February (44 vs 64 %) is due to the education of medical personnel to place patient labels such that the reading window is not obscured. The availability of new bottles from the manufacturer with a dedicated place for these labels could further improve the percentage of volumes read. Following our analysis of these volumes, we will target the hospital services for which the BC volumes are insufficient and inform the nurses. In addition, it has also been shown that monitoring the BC volume for paediatric bottles could improve diagnostic ability [11, 12].

Finally, we determined the rate of BC contamination. Among the positive BC samples, 27.2 % were probably contaminated. This results in additional laboratory tests, unnecessary antibiotic use and longer hospitalizations that increase patient care costs [13]. This rate might be higher, since we did not obtain the clinical context for all positive BCs. As previously reported, the contaminants were primarily coagulase-negative staphylococci [14, 15]. Hospital departments where the rate of contaminated positive BCs has been observed should be targeted to organize educational sessions.

To conclude, this study provides evidence that regular automated monitoring of several quality indicators can help to ensure the proper functioning of all steps necessary for the efficient diagnosis of bloodstream infections.
